# Temporal Ordering of Biomarkers in Dutch-Type Hereditary Cerebral Amyloid Angiopathy

**DOI:** 10.1161/STROKEAHA.123.044688

**Published:** 2024-03-06

**Authors:** Emma A. Koemans, Ingeborg Rasing, Sabine Voigt, Thijs W. van Harten, Reinier G.J. van der Zwet, Kanishk Kaushik, Manon R. Schipper, Nelleke van der Weerd, Erik W. van Zwet, Ellis S. van Etten, Matthias J.P. van Osch, Bea Kuiperij, Marcel M. Verbeek, Gisela M. Terwindt, Steven M. Greenberg, Marianne A.A. van Walderveen, Marieke J.H. Wermer

**Affiliations:** Departments of Neurology (E.A.K., I.R., S.V., R.G.J.v.d.Z., K.K., N.v.d.W., E.S.v.E., G.M.T., M.J.H.W.), Leiden University Medical Center, the Netherlands.; Radiology (S.V., T.W.v.H., M.R.S., M.J.v.P.O., M.A.A.v.W.), Leiden University Medical Center, the Netherlands.; Biostatistics (E.W.v.Z.), Leiden University Medical Center, the Netherlands.; Department Neurology and Genetics, Donders Institute for Brain, Cognition and Behaviour, Radboud University Medical Center, Nijmegen (B.K., M.M.V.).; Department of Neurology, University Medical Center Groningen, the Netherlands (M.J.H.W.).; J Philip Kistler Stroke Research Center, Department of Neurology, Massachusetts General Hospital, Harvard Medical School, Boston (S.M.G.).

**Keywords:** arteries, biomarkers, cerebral hemorrhage, magnetic resonance imaging, stroke

## Abstract

**BACKGROUND:**

The temporal ordering of biomarkers for cerebral amyloid angiopathy (CAA) is important for their use in trials and for the understanding of the pathological cascade of CAA. We investigated the presence and abnormality of the most common biomarkers in the largest (pre)symptomatic Dutch-type hereditary CAA (D-CAA) cohort to date.

**METHODS:**

We included cross-sectional data from participants with (pre)symptomatic D-CAA and controls without CAA. We investigated CAA-related cerebral small vessel disease markers on 3T-MRI, cerebrovascular reactivity with functional 7T-MRI (fMRI) and amyloid-β_40_ and amyloid-β_42_ levels in cerebrospinal fluid. We calculated frequencies and plotted biomarker abnormality according to age to form scatterplots.

**RESULTS:**

We included 68 participants with D-CAA (59% presymptomatic, mean age, 50 [range, 26–75] years; 53% women), 53 controls (mean age, 51 years; 42% women) for cerebrospinal fluid analysis and 36 controls (mean age, 53 years; 100% women) for fMRI analysis. Decreased cerebrospinal fluid amyloid-β_40_ and amyloid-β_42_ levels were the earliest biomarkers present: all D-CAA participants had lower levels of amyloid-β_40_ and amyloid-β_42_ compared with controls (youngest participant 30 years). Markers of nonhemorrhagic injury (>20 enlarged perivascular spaces in the centrum semiovale and white matter hyperintensities Fazekas score, ≥2, present in 83% [n=54]) and markers of impaired cerebrovascular reactivity (abnormal BOLD amplitude, time to peak and time to baseline, present in 56% [n=38]) were present from the age of 30 years. Finally, markers of hemorrhagic injury were present in 64% (n=41) and only appeared after the age of 41 years (first microbleeds and macrobleeds followed by cortical superficial siderosis).

**CONCLUSIONS:**

Our results suggest that amyloid biomarkers in cerebrospinal fluid are the first to become abnormal in CAA, followed by MRI biomarkers for cerebrovascular reactivity and nonhemorrhagic injury and lastly hemorrhagic injury. This temporal ordering probably reflects the pathological stages of CAA and should be taken into account when future therapeutic trials targeting specific stages are designed.

Cerebral amyloid angiopathy (CAA) is one of the most prevalent causes of intracerebral hemorrhage (ICH), cognitive decline, and dementia in the elderly.^1^ The disease is caused by a range of pathological changes following the deposition of the amyloid-β protein in the vessel wall of cortical and leptomeningeal arteries.^2^ The mechanisms that cause amyloid-β accumulation, the subsequent pathological cascade of brain damage and associated clinical symptoms are still not fully understood. Furthermore, it is unclear which biomarkers are present at different stages of this pathological cascade.

Research in the field of the pathophysiology underlying CAA development and progression is challenging. First, it is not possible to diagnose definite CAA during life, as this requires postmortem brain examination. Only a diagnosis of possible or probable CAA can be made during life based on the clinical and radiological Boston criteria 2.0.^3^ Second, most patients develop symptoms at an advanced age, when age-related brain changes and comorbidities may obscure or overlap with CAA-related pathology. This presentation in a relatively advanced stage of the disease furthermore complicates investigation of the earlier, presymptomatic phases.

One way to overcome these obstacles is to study hereditary CAA. Hereditary Dutch-type CAA (D-CAA) is one of the most common and best phenotyped hereditary variants of CAA.^4^ Pathological and biochemical changes, magnetic resonance imaging (MRI) findings and clinical symptoms are similar to sporadic CAA, except for an earlier onset and faster disease progression in D-CAA.^5^ DNA testing can identify mutation carriers with 100% certainty enabling research from early to advanced CAA. Studies in presymptomatic mutation carriers have identified several early biochemical and imaging biomarkers.^6–9^ The progression of these biomarkers over time is thought to reflect different disease stages similar to sporadic CAA and is essential information for future clinical trials.^10^

We investigated the presence and abnormality of currently known cerebrospinal fluid (CSF) and MRI biomarkers for CAA according to age in the largest (pre)symptomatic D-CAA cohort so far to increase our insight in their temporal ordering and interrelation, and to explore their consistency with our proposed pathophysiological framework for CAA.^10^

## METHODS

For this cross-sectional study, we included participants from the prospective D-CAA natural history study (AURORA, 2018-now) of the Leiden University Medical Center (LUMC).^11^ Symptomatic (defined as a history of ≥1symptomatic ICH) and presymptomatic mutation carriers with D-CAA aged >18 years were recruited via the clinic and outpatient clinic of the LUMC. We included participants who had the causal APP mutation for D-CAA or who had a history of symptomatic ICH on CT or MRI suspect for CAA and at least 1 first-degree relative with D-CAA. As part of the AURORA study, participants underwent yearly (presymptomatic participants every 2 years) research visits, which included a 3 and 7 T MRI, functional MRI (fMRI), blood and CSF collection, neurological and cognitive tests, and questionnaires on demographics and medical history, all performed on the same day. For the present study, the AURORA baseline data were used. The ethics committee of the LUMC approved the study and written informed consent was obtained from all participants before enrollment. For this study, we investigated the following biomarkers in CSF and on (f)MRI: amyloid-β_40_ and amyloid-β_42_, thought to reflect vascular amyloid deposition, measures of cerebrovascular reactivity at functional 7T-MRI (time to peak, time to baseline, and amplitude), measures of nonhemorrhagic injury (white matter hyperintensities [WMH] and enlarged perivascular spaces at the centrum semiovale) and hemorrhagic injury (microbleeds, macrobleeds, and cortical superficial siderosis [cSS]) at susceptibility weighted 3T-MRI. We chose these biomarkers as we assume that they reflect the steps of our recently published pathophysiological framework for CAA.^10^

Further information about our data set is available from the corresponding author upon reasonable request. This study was reported according to the STROBE guidelines (The Strengthening the Reporting of Observational Studies in Epidemiology).

### CSF and MRI Biomarkers

We investigated changes in CSF amyloid-β_40_ and amyloid-β_42_ levels in participants with D-CAA by comparing them with controls without CAA from the outpatient clinic of the Radboud University Medical Center.^7^ In the controls, CSF was obtained as part of a diagnostic workup during which central nervous system disorders were excluded.^12^ Controls were divided into age <55 years and age ≥55 years for comparison with (pre)symptomatic D-CAA groups, respectively. This cut-off point was chosen based on the mean age of the first symptomatic ICH in D-CAA.^13^ For details see the Supplemental Material.^12^

With 7T-fMRI we investigated visually stimulated blood-oxygen-level-dependent (BOLD) amplitude, time to peak (TTP), and time to baseline (TTB) as measures of cerebrovascular reactivity^8^ (T.W. van Harten, unpublished data, 2024). To investigate visually stimulated BOLD amplitude, TTP and TTB on 7T-fMRI in D-CAA we included control participants from the WHISPER study (White Matter Lesions in Young to Middle Aged Women with Stroke, Preeclampsia and Migraine: A 3 and 7 Tesla-Study).^8^ The WHISPER study is a 7T-MRI study of the LUMC that includes women between 40 and 60 years with a history of ischemic stroke and women without neurological disorders or systemic diseases associated with demyelinating or inflammatory white matter brain lesions. For the present study, we only included the control participants. WHISPER participants were retrieved via the outpatient clinic of the LUMC and via advertising and were scanned at the same scanner and using the same protocol as participants of the AURORA study. BOLD parameters from the high temporal resolution scans were obtained according to previously published methods (T.W. van Harten, unpublished data, 2024). For details see the Supplemental Material.

3T-MRI markers were scored according to the Standards for Reporting Vascular Changes on neuroimaging criteria 2.0. For details on the scoring of the markers, see the Supplemental Material.

### Statistics

Descriptive statistics were used to calculate frequency, medians, and means of baseline characteristics, CSF amyloid-β_40_ and amyloid-β_42_ levels, and MRI markers. Proportions were calculated with 95% CIs. Results were reported in temporal order by presenting those biomarkers first with the highest frequency at the youngest age of the participants. For details see the Supplemental Material. We created scatterplots illustrating amyloid-β_40_ and amyloid-β_42_ levels, BOLD parameters, peak width of skeletonized mean diffusivity, CAA cerebral small vessel disease (cSVD) score, and counts of cerebral microbleeds (CMB) and macrobleeds, and their correlation with age at time of lumbar puncture or MRI. For amyloid-β_40_ and amyloid-β_42_ levels and BOLD parameters, we estimated the age of divergence between D-CAA mutation carriers and controls, see the Supplemental Material. We considered a participant with D-CAA to have nonhemorrhagic injury on MRI if they had >20 enlarged perivascular spaces in the centrum semiovale (CSO-EPVS), >10 white matter spots and WMH Fazekas score of ≥2 (deep or periventricular) on MRI. Finally, we considered a participant with D-CAA to have signs of hemorrhagic injury if they had any CMB, macrobleeds, and cSS on MRI.

## RESULTS

**Figure 1.**
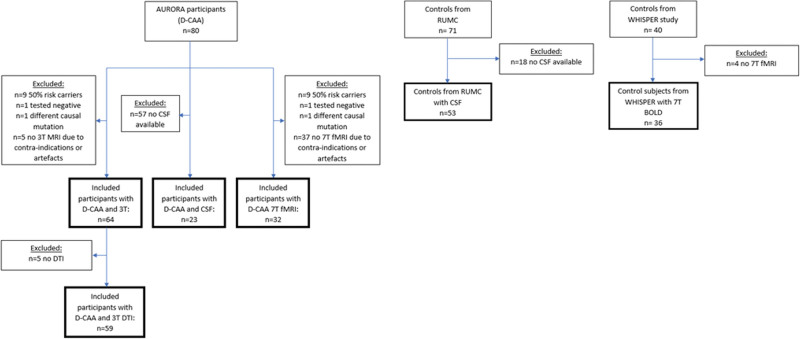
**Flowchart detailing participant inclusion.** Flowchart detailing participant inclusion from the AURORA and WHISPER studies and from the Radboud University Medical Center (RUMC). Fifty percent risk carries are participants without a clinical history of hereditary Dutch-type cerebral amyloid angiopathy (D-CAA) but with a positive family history of D-CAA in a first-degree relative, who did not undergo genetic testing.

We included 68 participants (age range, 26–75 years) with D-CAA; 37 presymptomatic (mean age, 43 years, 62% women) and 31 symptomatic (mean age, 59 years, 42% women). For baseline characteristics see Table S1, for a flowchart of participant inclusion see Figure 1. Of the 68 participants with D-CAA, 3T MRI was available in 64 (94%), CSF was available in 23 (34%), and 7T-fMRI was available in 32 (47%) participants.

### Frequency of Biomarkers

**Figure 2.**
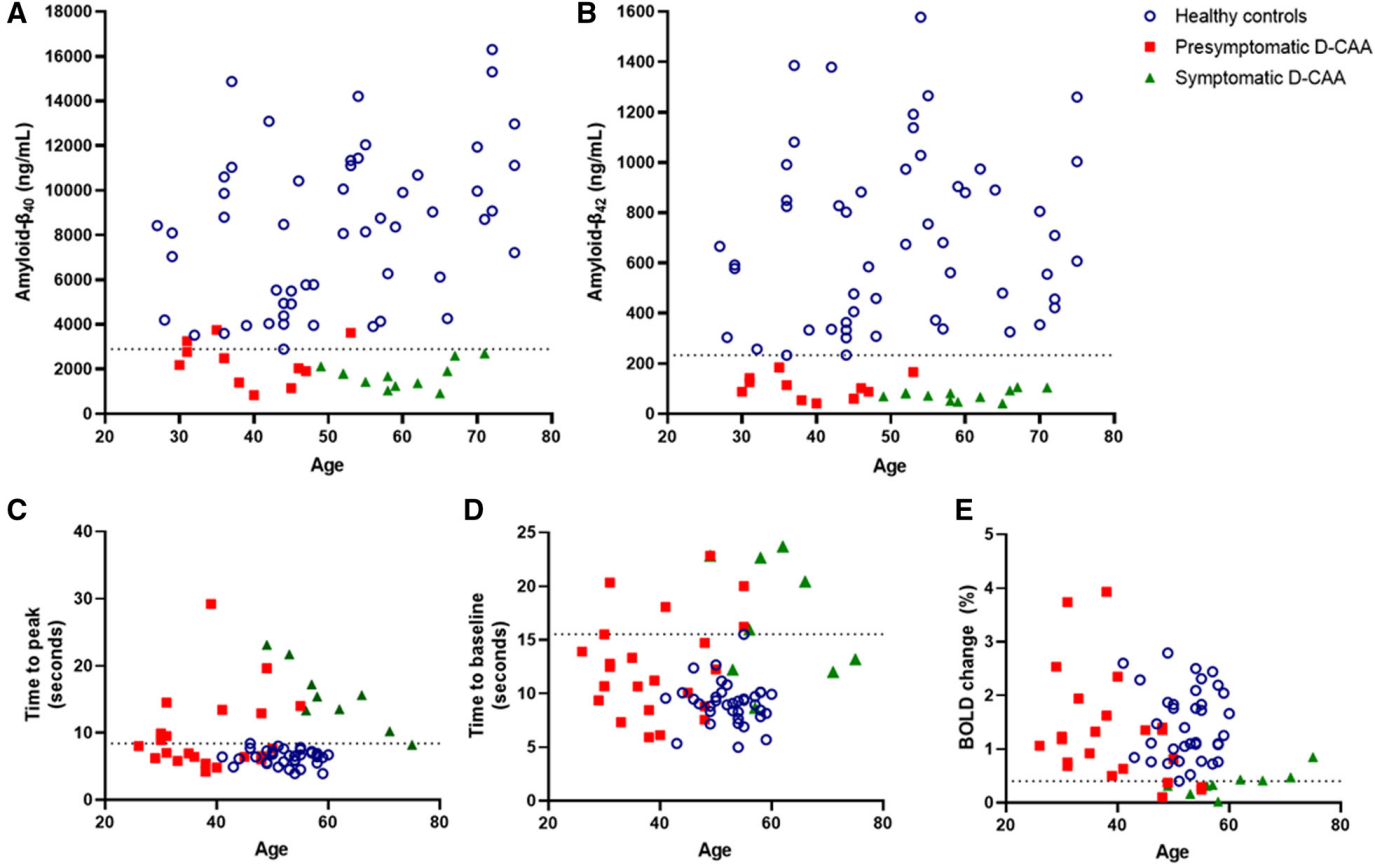
**Cerebrospinal fluid (CSF) amyloid-β_40_ and amyloid-β_42_ levels and blood-oxygen-level-dependent (BOLD) parameters at 7T-functional magnetic resonance imaging (7T-fMRI) in (pre)symptomatic participants with hereditary Dutch-type cerebral amyloid angiopathy (D-CAA) and controls.** In presymptomatic participants with D-CAA (n=23, red squares), symptomatic participants with D-CAA (n=9, green triangles) and healthy controls (n=36, blue circles). **A**, CSF amyloid-β_40_ levels (dotted line at cutoff 2889 ng/m). **B**, CSF amyloid-β_42_ levels (dotted line at cutoff 233 ng/mL) levels. **C**, BOLD 7T-fMRI time to peak in seconds (dotted line at cutoff 8.4 s). **D**, BOLD 7T-fMRI Time to baseline in seconds (dotted line at cutoff 15.5 s). **E**, BOLD 7T-fMRI amplitude in BOLD change (%; dotted line at cutoff 0.4% BOLD change).

Presymptomatic and symptomatic participants with D-CAA had lower median levels of amyloid-β_40_ and amyloid-β_42_ compared with (age-category matched) controls (Table 1). All participants with presymptomatic D-CAA except 3 had amyloid-β_40_ levels below the lowest control (cutoff, 2889 ng/mL, youngest participant below cutoff 30 years old; Figure 2A). Amyloid-β_42_ levels were lower in all participants with D-CAA compared with any of the controls (cutoff, 233 ng/mL, youngest participant below cutoff 30 years old; Figure 2B). The age of divergence for D-CAA mutation carriers and controls was −5.6 years for amyloid-β_40_ and −115.3 years for amyloid-β_42_ (Figure S1).

**Table 1.**
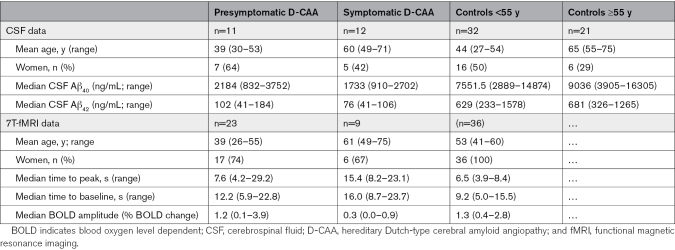
CSF Amyloid-β_40_ and β_42_ Markers and 7T-fMRI BOLD Parameters in (Pre)symptomatic D-CAA and Controls

Median TTP and TTB were higher, and BOLD amplitude lower, in presymptomatic and symptomatic participants with D-CAA compared with controls (Table 1; Figure 2). The youngest participant with D-CAA with a longer TTP compared with all controls was 30 years old, the youngest participant with D-CAA with a longer TTB compared with any of the controls was 31 years old. The youngest participant with D-CAA who had a lower BOLD amplitude compared with any of the controls was 48 years old. We determined the age of divergence for D-CAA mutation carriers and controls: for TTP this was 15.4 years, for TTB 19.2 years and for BOLD amplitude 31.5 years (Figure S1).

**Figure 3.**
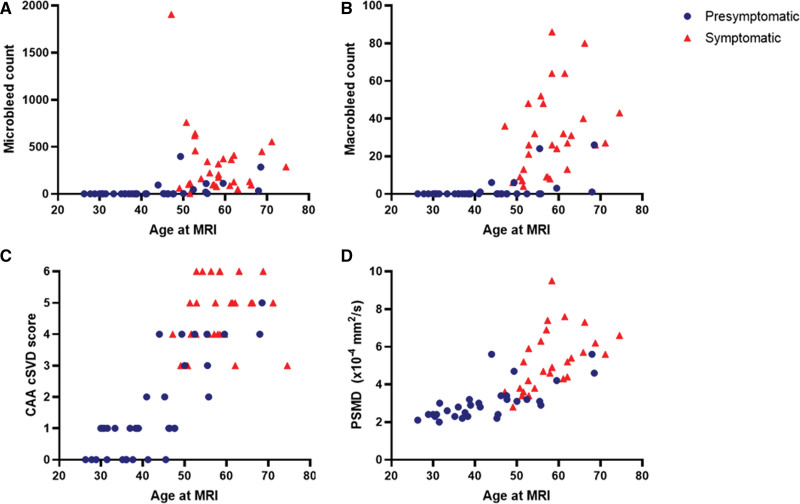
**Hemorrhage counts, cerebral amyloid angiopathy (CAA) cerebral small vessel disease (cSVD) sum scores and peak width of skeletonized mean diffusivity (PSMD) at 3T-magnetic resonance imaging (MRI) in (pre)symptomatic participants with hereditary Dutch-type CAA (D-CAA).** Microbleed count (**A**), macrobleed count (**B**), CAA cSVD score (**C**), and PSMD (**D**) in participants with presymptomatic D-CAA (n=35 for **A**, **B**, and **C**, n=31 for D; blue circles) and symptomatic D-CAA (n=29 for **A**, **B**, and **C**, n=28 for D; red triangles) according to age at MRI.

Markers of nonhemorrhagic injury (>20 CSO-EPVS, >10 white matter spots and Fazekas score, ≥2) were visible in 71% (95% CI 54–85) of presymptomatic, and 100% (95% CI, 88–100) of symptomatic participants (Table 2). The youngest participant with >20 CSO-EPVS was 30 years old, the youngest participant with >10 WMH spots was 31 years old and the youngest participant with WMH Fazekas score of ≥2 was 37 years old. Median peak width of skeletonized mean diffusivity scores were higher in symptomatic compared with presymptomatic participants (2.9 versus 5.1 mm^2^/s×10 to 4; Figure 3).

**Table 2.**
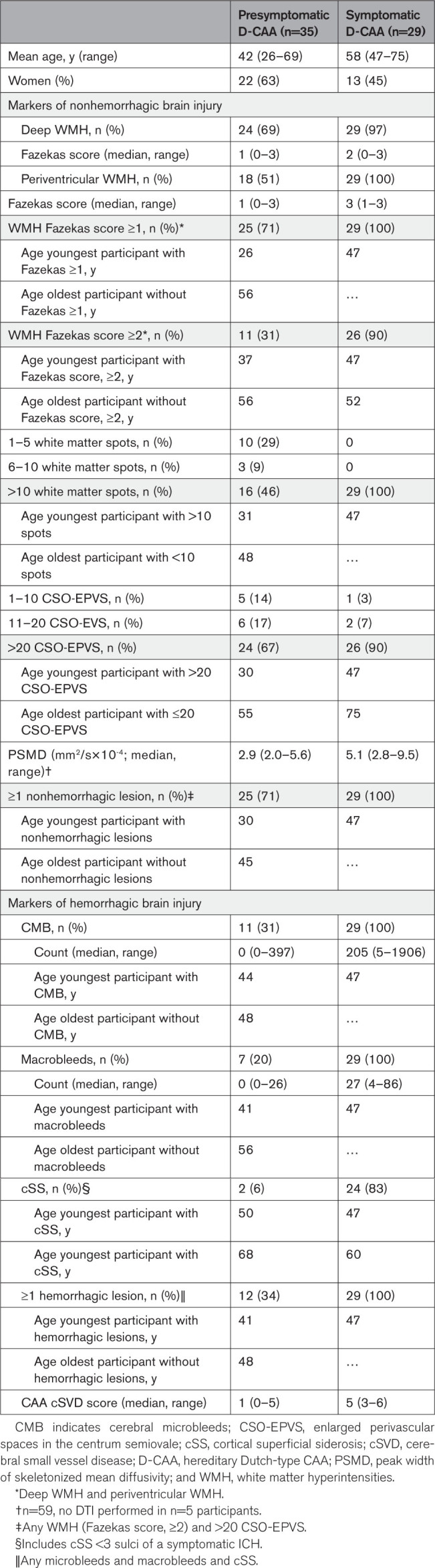
3T-MRI Markers in (Pre)symptomatic Participants With D-CAA

Markers of hemorrhagic injury (defined as presence of any CMB, macrobleeds, and cSS) were present in 34% (95% CI, 19–52) of presymptomatic and 100% (95% CI, 88–100) of symptomatic participants (Table 2). CMB and macrobleeds seemed to occur simultaneously (Table 2; Figure 3). The youngest participant with CMB was 44 years old, the youngest participant with macrobleeds was 41 years old. In our cohort, cSS seemed to occur the latest: the youngest presymptomatic participant with cSS was 50 years old and the youngest symptomatic participant with cSS was 47 years old (Table 2). The youngest participant with a marker reflecting hemorrhagic injury was ≈>11 years the youngest participant with a marker reflecting nonhemorrhagic injury (>20 CSO-EVS in a 30 year old participant, compared with a single macrobleed in a participant of 41 years old).

### Temporal Ordering of the Biomarkers

Levels of amyloid-β_40_ and amyloid-β_42_ in CSF were abnormal in 100% of the presymptomatic and symptomatic participants of our cohort (youngest participant with abnormal values was 30 years). Abnormal cerebrovascular reactivity was present in 44% of presymptomatic and 89% of symptomatic participants (youngest participant with abnormal values was 30 years). Thirteen participants had both available CSF and fMRI. Of those 13, 4 (all presymptomatic) had abnormal CSF amyloid-β_40_ and amyloid-β_42_ levels, while having normal cerebrovascular reactivity biomarkers. Sixteen (89%) participants with abnormal cerebrovascular reactivity also had signs of nonhemorrhagic brain injury on MRI. Fourteen (40%) of the presymptomatic participants with D-CAA had signs of nonhemorrhagic brain injury without hemorrhagic brain injury, and 9 (26%) did not have any signs of either nonhemorrhagic or hemorrhagic brain injury on MRI. All participants with signs of hemorrhagic injury had at least 1 feature of nonhemorrhagic injury (88% >20 CSO-EPVS, 95% >10% white matter spots, and 83% Fazekas score, ≥2). The youngest participant with a history of symptomatic ICH was 47 years old.

Based on these data, we speculated upon the temporal ordering of the biomarkers in this cohort, and suggest that in this cohort of participants with D-CAA levels of amyloid-β_40_ and amyloid-β_42_ in CSF were probably the first biomarkers to become abnormal, followed by markers of impaired cerebrovascular reactivity and nonhemorrhagic brain injury and lastly markers of hemorrhagic brain injury.

## DISCUSSION

In this cohort of participants with D-CAA, we found that CSF amyloid-β_40_ and amyloid-β_42_ levels seemed to be the earliest biomarkers to become abnormal with lower levels in all participants, followed by impaired vascular reactivity (expressed by increased TTP and TTB and decreased amplitude) and the occurrence of nonhemorrhagic brain injury (expressed by >20 CSO-EPVS, >10 white matter spots, and WMH Fazekas score of ≥2) in most participants from the age of 30 years on, and finally hemorrhagic brain injury (CMB and macrobleeds, followed by the development of cSS and lastly symptomatic ICH) appearing from the age of 40 years.

Our results seem to be in line with our recently proposed pathophysiological framework for CAA.^10^ In this framework, we proposed 4 steps: initial vascular amyloid deposition (step 1), alteration of cerebrovascular physiology (step 2), nonhemorrhagic brain injury (step 3), and finally appearance of hemorrhagic brain lesions (step 4).^10^ The framework was based on cohort studies in sporadic CAA and small studies in D-CAA, in which mostly 1 single biomarker at the time was investigated. Our current study includes the largest cohort of D-CAA mutation carriers thus far with novel data on a large set of different biomarkers all measured at the same time point. A limitation is that the D-CAA subjects were recruited from the prospective AURORA cohort, whereas controls are derived from 2 other studies; the RUMC study (Radboud University Medical Center; for CSF) and the WHISPER study (for 7T-fMRI). However, all data were collected prospectively, MRI scanners and protocols from the WHISPER and AURORA studies were identical as were CSF processing procedures in the LUMC and RUMC. In this way, we tried to minimize differences in MRI and sample handling to optimize comparison between the D-CAA and control cohorts. Definite validation of our previous proposed framework would require longitudinal data of both a large group of participants with D-CAA and controls.

We found that the earliest biomarkers for CAA pathology are abnormal in mutation carriers in their mid-20s and early thirties, decades before the age at which in general the first ICH occurs (the youngest age of onset of first ICH in our cohort was 47 years). We did not enroll children/adolescents in our study and, therefore, we could not investigate the exact age at which the amyloid levels in CSF and the vasoreactivity start to become abnormal.

Although in the cohort the biomarkers seemed to occur in a fixed temporal ordering, we found a striking variability in the timing of which the different biomarkers become abnormal in individual patients. As our data are cross-sectional, it is unclear how this variation evolves over time. It is for example unclear whether patients who develop abnormal biomarkers relatively early in life are also the first ones to develop the biomarkers associated with the advanced stages of CAA. This individual variability in clinical and radiological phenotype is also present in sporadic CAA, and suggests that there are (risk) factors that influence disease onset and course. Possible influential factors are hypertension, the use of antithrombotic/coagulating drugs and *APOE* genotype, although the role of this last factor has not been fully elucidated in D-CAA.^14–17^ Apart from these known factors, several other, (epi)genetic, biological, lifestyle, or environmental factors might exist that influence D-CAA disease course.^18^

Our results have important implications for future clinical research. The stepwise progression of the biomarkers as suggested in our current study can help to identify timing and type of candidate treatments for future clinical trials in CAA. Our results suggest that in CAA there is significant lag time of 20 to 30 years between the first CSF and MRI signs of the disease and the development of clinical symptoms. This time lag offers a window of opportunity for both prevention and disease-modifying drugs: in the early nonhemorrhagic phases of the disease, tissue injury could hypothetically still be prevented, whereas in the late hemorrhagic stage disease progression or onset of ICH can be delayed but cerebral injury will probably be irreversible. Treatment in the late stage mainly has to target triggers of ICH. Inclusion criteria for trials should take the different steps and their associated biomarkers into account to time when in the disease process the drug of interest would take action and which disease mechanisms would be targeted.

Our results on the interrelation of biomarkers may also have implications for the diagnosis of CAA (mimics). We showed that all patients with D-CAA who had hemorrhagic markers also had nonhemorrhagic markers on their MRI. Although the new Boston 2.0 criteria emphasize the importance of nonhemorrhagic markers, the diagnosis possible CAA can still be based on the presence of clinical symptoms and hemorrhagic markers only. Our findings, however, suggest that it is unlikely to have no nonhemorrhagic signs in the hemorrhagic stage of CAA. Therefore, in patients with suspicion of CAA based on CMB, macrobleeds, or cSS only without the presence of WMH or CSO-EPVS, alternative diagnoses such as multiple small cavernomas or reversible vasoconstriction syndrome should be considered. In our cohort, cSS was the last biomarker to become abnormal, suggesting that it is a sign of late-stage CAA. cSS is strongly related to leptomeningeal vessel remodeling (Vonsattel grade III: concentric splitting of the vessel wall) at histopathology, as opposed to CMB, which are related to cortico-subcortical vessel remodeling (Vonsattel grade IV: replacement of the arteriolar wall with fibrinoid material).^19,20^ It is possible that leptomeningeal vessels are affected later in the disease, or that grade III occurs in later, more severe stages than grade IV.^19^

Our study has several limitations. First, we used a cross-sectional study design: data on the longitudinal progression of biomarkers in individual patients are not yet available in our cohort and the exact time points of the first appearance of markers could, therefore, not be assessed. Within the D-CAA cohort, there was substantial sample loss, especially regarding the CSF data, which complicates interpretation. Second, we use categorical variables to measure WMH (Fazekas score and multispot pattern), presence of multiple white matter spots and CSO-EPVS which, are subject to ceiling effects.^21,22^ Furthermore, our cutoff for CSO-EPVS (>20), spots (>10) and WMH (Fazekas score, ≥2) remain semiartificial.^3^ Unfortunately, quantitative measures for CSO-EPVS, spots, and WMH are not yet available for our cohort. In the future, the availability of quantitative measures might enable a better distinction of differences in temporal ordering of the nonhemorrhagic steps of the cascade. For peak width of skeletonized mean diffusivity, a quantitative marker for diffuse brain injury, we unfortunately had no available control participants to investigate abnormality. However, compared with data from previously published, population-based cohorts, the peak width of skeletonized mean diffusivity values in our participants with CAA seemed to be on average higher compared with healthy, age-matched controls (Figure 3D).^23–25^ A third limitation is that due to the inclusion of only women in the WHISPER study we were not able to include male control subjects for the 7T-fMRI data. Another limitation is that although CSF amyloid-β_40/42_ levels and BOLD fMRI parameters are present in the early phases of D-CAA, there is no universal established cutoff for when the levels of these markers are abnormal. Therefore, they are not suitable for determining disease stages in individual patients.^7,8^ By using a cutoff based on the lowest threshold of controls without CAA, we could only infer reference-based and no absolute abnormality. The cut-off points requiring a threshold lower (or higher) than all controls, however was strict by definition. Finally, although D-CAA is a useful model for sporadic CAA, there are also differences that might limit generalizability. All D-CAA mutation carriers eventually develop a symptomatic ICH. In sporadic CAA, there seems to be more variation in different pathophysiological and clinical phenotypes, perhaps due to vascular or Alzheimer comorbidity, and not all patients suffer from symptomatic ICH.^18^ Strengths of our current study are the relatively large cohort of participants with this pure, hereditary form of CAA, and the use of different modalities for investigating biomarker abnormality (CSF, MRI, and fMRI), all obtained on the same day. Our results have important implications for future clinical trial design, aiding in the identification and timing of candidates for disease-modifying treatments and the choice for the appropriate biomarkers to monitor treatment effect.

## ARTICLE INFORMATION

### Acknowledgments

The authors would like to thank and acknowledge N. Vlegels for her work on the peak width of skeletonized mean diffusivity data, H.J.A. van Os for his work on the WHISPER study, and A. de Kort for her work on the cerebrospinal fluid data.

### Sources of Funding

The Netherlands Heart Foundation grant 2016T086 to Dr Wermer, the Dutch CAA foundation. BIONIC project (no 733050822, ZonMW). Memorabel grant, as part of the Dutch national Deltaplan for Dementia. The BIONIC project is a consortium of Radboudumc, LUMC, ADX-Neurosciences, and University of Rhode Island. The funding agencies had no role in the design or conduct of the study.

### Disclosures

Dr van der Zwet and M. R. Schipper report support from the TRACK D-CAA consortium (Alnylam, Biogen, the Dutch CAA foundation, Vereniging HCHWA-D, researchers from Leiden, Boston, Perth). Dr van Osch reports support by a Nederlandse Organisatie voor Wetenschappelijk Onderzoek (NWO)-VICI grant (016.160.351) and a NWO-Human Measurement Models 2.0 grant (18969), European Community, the Dutch Heart Foundation, the Dutch Brain Foundation VSNU Netherlands and the TRACK D-CAA consortium. Dr Verbeek reports independent support from ZonMw (09120012110098, 10510032120006, and 10510032120003), the Galen and Hilary Weston Foundation (NR170024), Maag-Lever-Darm Stichting (21-05), Parkinson NL (P2021-18), Stichting Alkemade-Keuls, National Institutes of Health (5R01NS104147). Dr Terwindt reports independent support from de NWO, European Community, the Dutch Heart Foundation, the Dutch Brain Foundation, and the Dutch CAA foundation, compensation from Teva Pharmaceutical Industries, H. Lundbeck A S, Novartis Pharma, and Eli Lilly and Company for consultant services. Dr Greenberg reports independent support from the US National Institutes of Health. Dr Wermer independent support from the NWO ZonMw (VIDI grant 91717337), the Netherlands Heart Foundation (2016T86). The other authors report no conflicts.

### Supplemental Material

STROBE Checklist

Supplemental Materials and Methods

Table S1

Figure S1

References ^26–31^

## Supplementary Material


