# Supplementary material for: Temporal Ordering of Biomarkers in Dutch-Type Hereditary Cerebral Amyloid Angiopathy
Source: Stroke. 2024 Mar 6;55(4):954–62. doi: 10.1161/STROKEAHA.123.044688 (PMC10962436; doi:10.1161/STROKEAHA.123.044688)
Supplement: Supplementary file 1 [file str-55-0954-s001.pdf]

SUPPLEMENTAL MATERIAL

Supplemental methods

CSF acquisition

The CSF samples from the healthy control group of the RUMC cohort obtained as part of diagnostic work-up were coded and used with full consent from the participants. CSF samples were obtained from AURORA participants and from the controls of the RUMC under standardized conditions.[12] CSF amyloid-β_{40} and amyloid-β_{42} levels were quantified at the RUMC using Lumipulse® G fully automated immunoassays (Fujirebio, Ghent, Belgium).

Image acquisition 3T

AURORA participants were scanned using a whole body 3 Tesla (3T) MRI system (Philips Healthcare, Best, The Netherlands). The following sequences were performed using a standard 32-channel head coil: Three-dimensional T1 weighted images (3DT1) (repetition time (TR)/ echo time (TE) = 9.7/4.6ms, flip angle 7 degrees, 130 slices with no interslice gap, field of view (FOV) 217x172x156mm, voxel size 1.2x1.2x1.2mm), T2 weighted images (TR/TE = 4744/80ms, flip angle 90 degrees, 48 slices with no interslice gap, FOV 220x176x144mm, voxel size 0.5x0.6x3mm). Three dimensional Fluid Attenuated Inversion Recovery (FLAIR) images (TR/TE = 4800/280ms, inversion time (TI) 1650ms, 321 slices with no interslice gap, FOV of 250x250x180mm, voxel size 1.1x1.1x0.6 mm), susceptibility weighted images (SWI) (TR/TE = 31/7.2ms, flip angle 17 degrees, 130 slices and an FOV of 230x190x130 mm, voxel size of 0.6x0.6x1mm). Diffusion images (DTI) (TR/TE = 8194/76 ms, voxel size 1.72x1.72x2.5 mm, flip angle = 90 degrees, 48 slices and FOV of 220x220x120mm, 45 gradient directions with a b-value of 1200 s/mm2 and one baseline image with b-value 0 s/mm2).

Image analysis 3T

The following 3T-MRI markers were scored according to the Standards for Reporting Vascular Changes on neuroimaging (STRIVE) criteria 2.0: cerebral microbleeds (CMB), macrobleeds, cortical superficial siderosis (cSS), CSO-EPVS, periventricular and deep WMH.[26] CMB count, macrobleed count and cSS focality were scored on susceptibility weighted images (SWI).[27] CSO-EPVS were scored on T2-weighted images and classified into the following categories; no EPVS, 1-10 EPVS, 11-20 EPVS, 21-40 EPVS, >40 EPVS.[22] Deep and periventricular WMH were graded with the Fazekas score on FLAIR images.[23] On each FLAIR image white matter spots were counted (defined as small circular or ovoid hyperintense lesions in the bilateral subcortical white matter), and presence of a multi-spot pattern (defined as >10 white matter spots) was assessed.[3] Based on the diffusion tensor imaging (DTI), peak width of skeletonized mean diffusivity (PSMD) was calculated according to previously published methods.[28] PSMD is a marker used for the interpretation of white matter integrity, and is related to neurovascular injury and cognitive performance in CAA.[28,29] For each participant the CAA cerebral small vessel disease (cSVD) score was calculated according to previously published methods using the following markers; lobar CMB, cSS, CSO-EPVS, and WMH.[30] All structural MRI markers were scored by one observer (E.A.K, >5 years of experience in the field), apart from white matter spots, which were scored by R.v.d.Z (2 years of experience in the field). In case of doubt, findings were discussed with a third observer with >15 years of experience in the field (M.A.A.v.W.) for the final decision.

Image acquisition 7T

Participants of the AURORA study and the WHISPER study underwent MRI scanning on a on a whole body human 7T MR system (Philips, Best, the Netherlands). For AURORA participants this 7T MRI was performed on the same day as the 3T and lumbar puncture. A

quadrature transmit and 32-channel receive head coil (Nova Medical, Wilmington, MA, USA) was used and all participants were scanned according to a protocol which has been published in previous studies.[30,31] (T.W. van Harten, MSc, unpublished data, 2023) The protocol included a 3DT1 weighted scan (scan parameters: repetition time (TR) 4.146 ms, echo time (TE) 1.86 ms, flip angle 7°, field of view (FOV) 240 x 255 x 246 mm, 250 slices, slice thickness 0.9 mm, matrix size 288 x 288, and scan duration 142 s) and a coronal single slice high temporal resolution fMRI scan through the occipital cortex (scan parameters: TR 100 ms, TE 22 ms, flip angle 20°, FOV 100 x 141 mm, slice thickness 1.5 mm, matrix size 128 x 128, 3360 dynamics, and scan duration 336 s) with a visual stimulus of seven 48s blocks consisting of 3 seconds flashing black and white radial checkerboard pattern at 8 Hz, followed by 45 seconds of grey screen.

Statistics

We considered a participant with D-CAA to have abnormal levels of amyloid-β_{40} and/or amyloid-β_{42} if these were lower than in any of the controls. Similar, we considered a participant with D-CAA to have abnormal cerebrovascular reactivity if TTP and/or TTB were higher and/or amplitude lower than the highest/lowest control. We estimated age of divergence between D-CAA mutation carriers and controls, according to previously published methods: we used linear regression lines and estimated confidence bands for the lines. Age of divergence was then determined as the point where the 95% credible intervals of the difference distribution did not overlap.[32]

Table S1. Baseline characteristics of the participants with D-CAA.

	All (n=68)	Presymptomatic D-CAA (n=37)	Symptomatic D-CAA (n=31)
Age in years (mean, range)	50 (26-75)	43 (26-69)	59 (47-75)
Women (n, %)	36 (53)	23 (62)	13 (42)
Symptomatic ICH (n,%)	31 (47)	0	31 (100)
Hypertension (n, %)	16 (24)	8 (22)	8 (26)
Hypercholesterolemia (n, %)	15 (22)*	4 (11)	11 (36)[a]
Diabetes mellitus type 2 (n, %)	3 (4)	1 (3)	2 (7)
Smoking, ever (n, %)†	43 (63)	23 (62)	20 (65)

D-CAA: hereditary Dutch-type CAA. ICH: intracerebral hemorrhage.

**Data missing in n=1 participant.* †*Defined as having smoked ≥1 cigarette/day for the duration of at least one year.*

Figure S1. Ages of divergence between D-CAA mutation carriers and controls for CSF and fMRI BOLD parameters

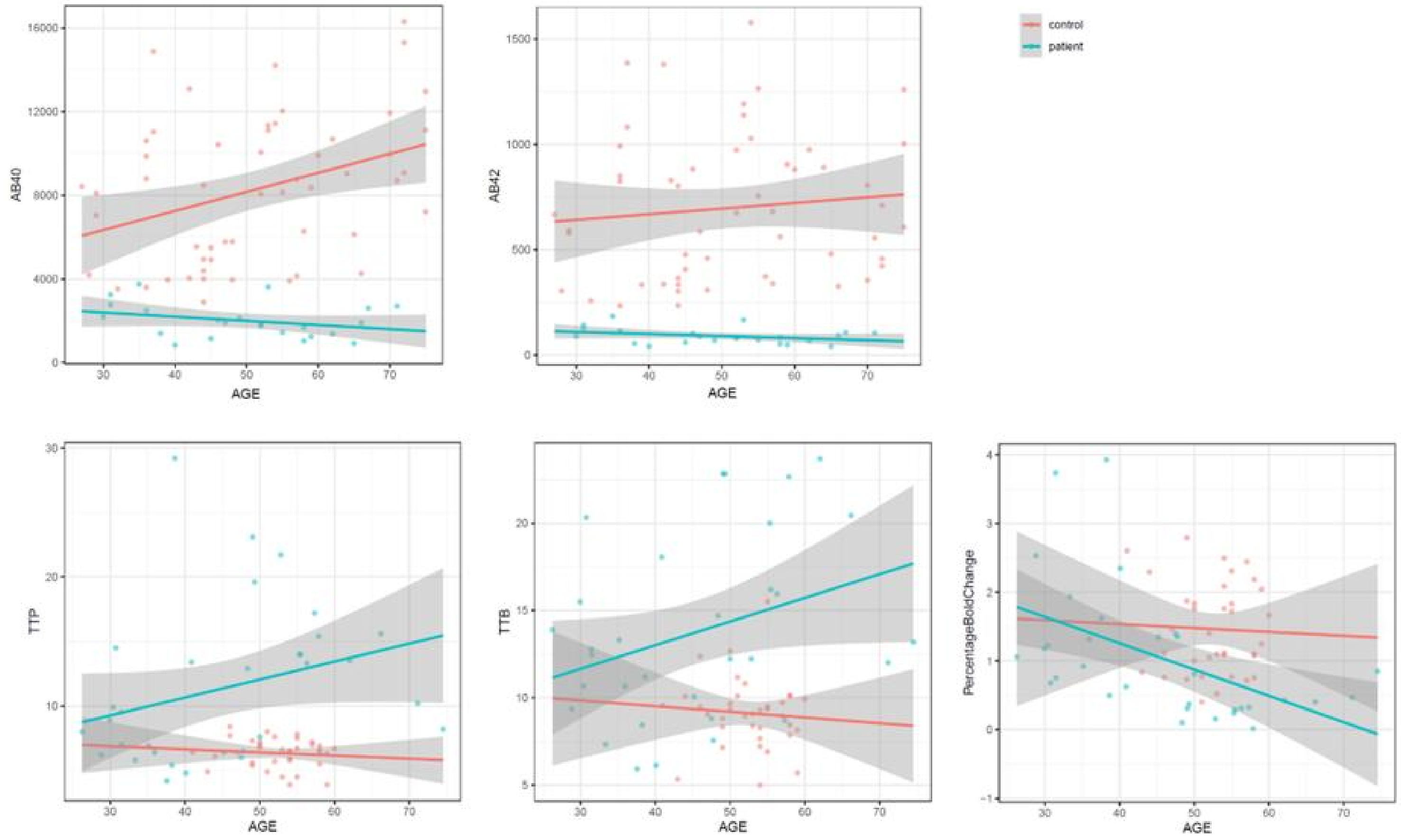

Age of convergence for patients and controls calculated by determining the intersection point of the 95% confidence intervals. Determined for ***A****. amyloid-β_{40} levels in ng/ml, age of convergence -5.6 years.* ***B****. amyloid-β_{42} levels in ng/ml, age of convergence -115.3 years.* ***C****. Time to peak in seconds, age of convergence 15.4 years.* ***D****. Time to baseline in seconds, age of convergence 19.2 years.* ***E****. Amplitude in BOLD change %, age of convergence 31.5 years*
